# An extension of the coevolution theory of the origin of the genetic code

**DOI:** 10.1186/1745-6150-3-37

**Published:** 2008-09-05

**Authors:** Massimo Di Giulio

**Affiliations:** 1 Laboratory for Molecular Evolution, Institute of Genetics and Biophysics 'Adriano Buzzati Traverso', CNR, Via P. Castellino, 111, 80131 Naples, Napoli, Italy

## Abstract

**Background:**

The coevolution theory of the origin of the genetic code suggests that the genetic code is an imprint of the biosynthetic relationships between amino acids. However, this theory does not seem to attribute a role to the biosynthetic relationships between the earliest amino acids that evolved along the pathways of energetic metabolism. As a result, the coevolution theory is unable to clearly define the very earliest phases of genetic code origin. In order to remove this difficulty, I here suggest an extension of the coevolution theory that attributes a crucial role to the first amino acids that evolved along these biosynthetic pathways and to their biosynthetic relationships, even when defined by the non-amino acid molecules that are their precursors.

**Results:**

It is re-observed that the first amino acids to evolve along these biosynthetic pathways are predominantly those codified by codons of the type GNN, and this observation is found to be statistically significant. Furthermore, the close biosynthetic relationships between the sibling amino acids Ala-Ser, Ser-Gly, Asp-Glu, and Ala-Val are not random in the genetic code table and reinforce the hypothesis that the biosynthetic relationships between these six amino acids played a crucial role in defining the very earliest phases of genetic code origin.

**Conclusion:**

All this leads to the hypothesis that there existed a code, GNS, reflecting the biosynthetic relationships between these six amino acids which, as it defines the very earliest phases of genetic code origin, removes the main difficulty of the coevolution theory. Furthermore, it is here discussed how this code might have naturally led to the code codifying only for the domains of the codons of precursor amino acids, as predicted by the coevolution theory. Finally, the hypothesis here suggested also removes other problems of the coevolution theory, such as the existence for certain pairs of amino acids with an unclear biosynthetic relationship between the precursor and product amino acids and the collocation of Ala between the amino acids Val and Leu belonging to the pyruvate biosynthetic family, which the coevolution theory considered as belonging to different biosyntheses.

**Reviewers:**

This article was reviewed by Rob Knight, Paul Higgs (nominated by Laura Landweber), and Eugene Koonin.

## Background

### Why the genetic code originated

There are two completely different interpretations on why the genetic code might have originated. The first is obtained by means of an extreme interpretation of the stereochemical hypothesis of genetic code origin which suggests that the genetic code originated because its organisation is somehow constrained by the stereochemical relationships between codons or anticodons and amino acids. This extreme interpretation seems totally absurd to me. The second interpretation that I am aware of has to do with the origin of peptidyl-tRNA: the key intermediate in the origin of protein synthesis.

Peptidyl-tRNA has no function per se, but in some models it has been assumed that the entire catalysis of the protocell was originally performed by this intermediate [1-4]. Its origin might therefore have been determined by interactions between covalent complexes of peptide and RNA (peptide-RNAs) and these interactions might have constituted one of the most elementary forms of protein synthesis [3,4]. This model shows that the interactions between peptide-RNAs must, at a certain evolutionary stage, have been directed by a template (pre-mRNA) which must have originally codified only the succession of interactions between peptide-RNAs [4]. This pre-mRNA is the most ancestral form of mRNA imaginable [4]. Finally, the evolution of these pre-mRNAs must have resulted in an mRNA codifying only for a limited number of amino acids [4]. This is the phase that defines the very origin of the genetic code. Clearly this is an historic interpretation of genetic code origin that is completely different from the deterministic one given by the stereochemical theory.

What is particularly important as far as this paper is concerned is that the evolution of these pre-mRNAs into mRNAs was characterised by a progressive refinement of the interactions of the peptide-RNAs on the pre-mRNA templates and this refinement seems to have been made possible only when peptide-RNAs were transformed into amino acid-pre-tRNAs [4]. This is because there might have only been the modification, residue by residue, performed by the amino acid-pre-tRNAs on the evolving proteins that might lead to the complete specification of their sequences, and which made possible the birth of an mRNA proper but with codification limited to just a few amino acids [4]. As will become clear in the following, I maintain that these amino acid-pre-tRNAs came directly from the biosynthetic pathways of the first six amino acids evolving along the biosynthetic pathways of energetic metabolism and that they were the first amino acids to be codified on these still evolving mRNAs.

### The biosynthetic relationships between amino acids are closely linked to the organisation of the genetic code

Ever since the genetic code was first deciphered, it has been observed that the biosynthetic relationships between amino acids are linked to the organisation of the genetic code. Indeed, Nirenberg et al. [5] acknowledged the existence of a relationship between amino acids of a similar biosynthetic origin and the codons specifying those amino acids. Although the examples of biosynthetic relationships reported by Nirenberg et al. [5] contain some inaccuracies, the authors were the first to suggest that the genetic code's evolutionary development might have been defined by the amino acids' biosyntheses. Jukes [6] also noted that some amino acids take part in the biosynthesis of other amino acids, such as serine which plays a part in the biosynthesis of tryptophan. However, these seemed to be isolated and not totally clear observations and Jukes [6] did not believe they could be generalised for the entire genetic code. Pelc [7] recognised that biosynthetic conversions between amino acids might have had an important role in defining the genetic code. However, it was Dillon [8] who, above all, suggested a metabolic model for the origin of the genetic code, although this author suggested amino acid biosyntheses that are only partly linked to those existing in living organisms. It was Wong [9] who fully recognised the importance, for the evolution of the genetic code, of the biosynthetic relationships between amino acids as they take place in actual organisms, suggesting what is now known as the coevolution theory of genetic code origin. This theory suggests that the genetic code is primarily an imprint of the biosynthetic pathways forming amino acids [9]. Consequently the evolution of the genetic code could be clarified on the basis of the precursor-product relationships between amino acids in their biosyntheses [9]. In other words, this theory suggests that only few amino acids (precursors) were codified in the genetic code; as other amino acids (products) developed from these, part of the codon domain of precursor amino acids was ceded to product amino acids [9]. Therefore, according to this theory, the genetic code might represent an evolutionary map of the biosynthetic relationships between amino acids [9].

**Figure 1:**
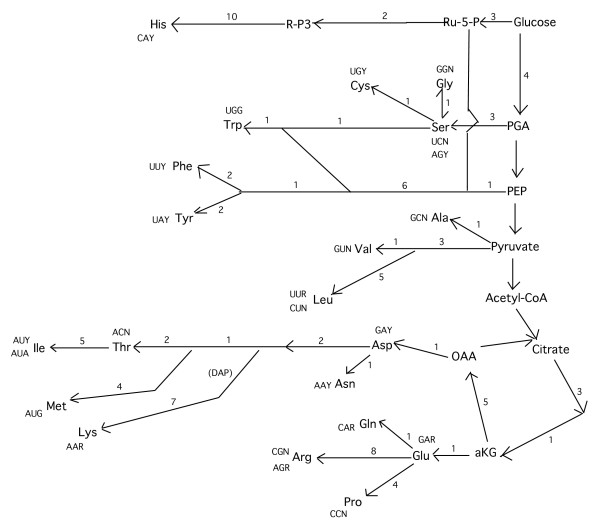
**Biosynthetic relationships between amino acids, as defined by their biosyntheses and their relationships with the glycolytic pathway and the citric acid cycle.** The figure was taken from Taylor and Coates [11] with a few modifications. The numbers indicate the biosynthetic steps. DAP = diaminopimelic pathway, aKG = alpha-ketoglutarate, OOA = oxalacetic acid, PEP = phosphoenolpyruvate, PGA = phosphoglycerate, R-P3 = 5-phosphoribosylpyrophosphate, Ru-5-P = ribulose-5-phosphate. The other abbreviations are standard.

While Wong [9] highlighted the precursor-product relationships between amino acids and their crucial role in defining the organisation of the genetic code, Miseta [10] clearly identified that the non-amino acid molecules that were precursors of amino acids might have been able to play an important role in organising the genetic code. Miseta [10] suggested the idea of an intimate relationship between molecules, the intermediates of glucose degradation, as precursors of precursor amino acids, and the organisation of the genetic code. This observation is also analysed by Taylor and Coates [11] who showed the relationship between the glycolytic pathway, the citric acid cycle, the biosyntheses of amino acids and the genetic code (Fig. 1) and, in particular, they point out that (i) all the amino acids that are members of a biosynthetic family tend to have codons with the same first base (Fig. 1) and (ii) that the five amino acids codified by GNN codons are found in four biosynthetic pathways close to or at the beginning of the pathway head (Fig. 1)[11]. More recently, Davis [12,13] has provided evidence that tRNAs descending from a common ancestor were adaptors of amino acids synthesised by a common precursor and he also discusses the biosynthetic families of amino acids, suggesting their importance in genetic code origin.

However, there have also been authors who have suggested that some aspects of the biosynthetic relationships between amino acids were not important in genetic code origin [14,15]. In particular, Ronneberg et al. [14] criticise the coevolution theory above all because some pairs of amino acids used by this theory do not seem to be in a clear precursor-product amino acid relationship, although, more generally, they recognise that amino acids in a biosynthetic relationship tend to have codons with the same first base [14]. Di Giulio [16] responded to the criticisms made by Ronneberg et al [14] and, in particular, made numerous observations in favour of the coevolution theory. There has also been evidence indicating that the five families of amino acids, defined in accordance with a single amino acid precursor or a non-amino acid precursor, should have been randomly observed in the genetic code with a probability of 6 × 10^-5 ^[17]. This indicates that the biosynthetic relationships between amino acids were fundamental in organising the genetic code.

Finally, if we consider that other works have been carried out on the importance of biosynthetic relationships between amino acids and the genetic code [18-39], we come to the conclusion that there can no longer be any doubts on the hypothesis that the origin of the organisation of the genetic code was affected by the biosynthetic pathways of amino acids.

## Results

### The extended coevolution theory

In order to eliminate some criticisms on certain pairs of amino acids that are in an unclear precursor-product relationship [14,16] and, above all, to provide a more complete description of the very earliest phases of genetic code origin, I have been forced to suggest the following theory. This theory, which can be called the 'extended coevolution theory' as it is simply an extension or a generalisation of Wong's coevolution theory [9], states that:

*"The genetic code is simply an imprint of the biosynthetic relationships between amino acids, even when defined by the non-amino acid molecules that are the precursors of some amino acids, i.e. that the organisation of the genetic code must only reflect the biosynthetic proximity between amino acids in the various stages of evolution of their biosynthetic pathways. This happened because the ancestral biosynthetic pathways took place on tRNA-like molecules and thus enabled a coevolution between these pathways and the organisation of the genetic code through the concession of tRNA-like molecules between biosynthetically close amino acids, which made possible the transfer of codons from one amino acid to another, while mRNA evolved, with the consequence that amino acids with correlated biosyntheses have contiguous codons in the genetic code"*.

This theory, which in a contracted and informal form has already been suggested [16], can be tested and all the evidence in favour of the coevolution theory is also in favour of the extended coevolution theory. The key point on which the two theories disagree regards the predictions on the earliest phases of genetic code origin, which are not well defined for the coevolution theory [9,40] while, for the extended coevolution theory their traces should be present in the biosynthetic relationships between amino acids that are precursors of other amino acids and the non-amino acid molecules that are precursors of precursor amino acids.

As shown in the following section, this main prediction of the extended coevolution theory seems to be corroborated by the observations.

### The main prediction of the extended coevolution theory seems to be corroborated

According to the predictions of the coevolution theory, the codon concession mechanism between amino acids in a precursor-product relationship was based on tRNA-like molecules on which the theory hypothesises that biosynthetic transformations between amino acids take place [9]. Surprisingly, this prediction is confirmed by the existence of molecular fossils [33] representing the vestiges of these pathways (Tab. 1) hypothesised by the coevolution theory [9,19-21]. Although these biosynthetic transformations took place in accordance with the coevolution theory, only among the amino acids in a precursor-product relationship [9] is there no a priori reason why this should have taken place only between amino acids [28,31]. The coevolution theory seems to imply that all metabolism took place at that time on tRNA-like molecules [28,31] or, at least, that the entire metabolism of amino acids took place on these molecules. This view, i.e. that metabolism took place on tRNA-like molecules, has been hypothesised by other authors following arguments that might be totally different from those used here [41-43].

Therefore, if the metabolism of amino acids took place on tRNA-like molecules when the genetic code originated, the structure of the genetic code must contain traces linking the very earliest phases of genetic code origin to the biosynthetic relationships between the first amino acids to enter the code and the non-amino acid molecules that were their precursors. This is because the very first amino acids that entered the genetic code and had non-amino acid molecules as their precursors, did so, as suggested by the extended coevolution theory, using the same mechanism employed by the pairs of amino acids in a precursor-product relationship, i.e. exploiting the hypothetical existence of the biosynthetic pathways on the tRNA-like molecules that triggered the origin of the genetic code. This is the main prediction of the extended coevolution theory and how it differentiates the latter from the coevolution theory.

**Figure 2:**
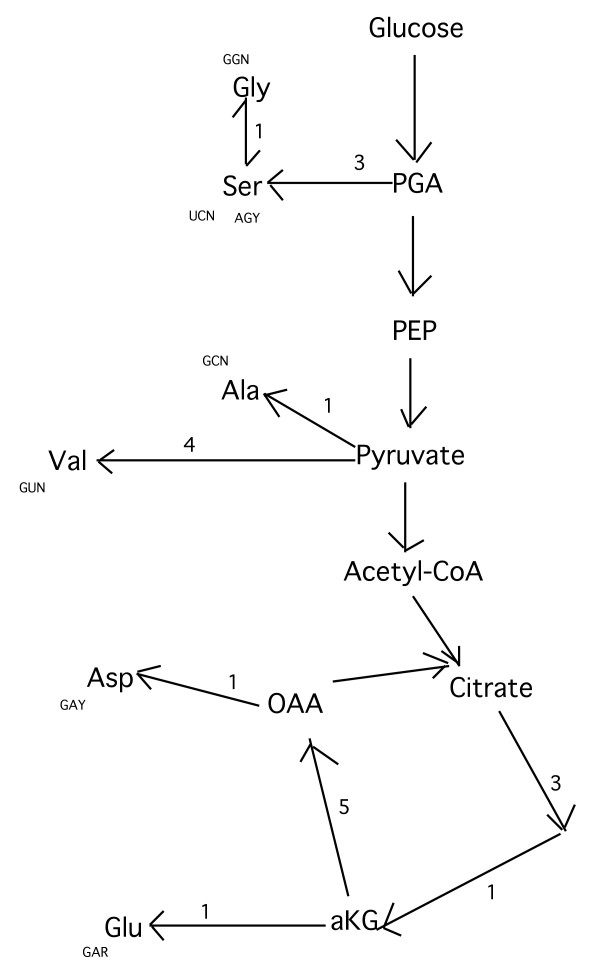
**Biosynthetic relationships between amino acids and their precursor non-amino acid molecules, as defined in a particular stage of the evolution of the biosynthetic pathways of amino acids.** With the sole exception of proline, these are also the amino acids that first appear in a study on the temporal origin of the appearance of amino acids [54]. See Fig. 1 for further information.

Fig. 2 reports the biosynthetic relationships between amino acids that presumably first originated from the glycolytic pathway and Krebs' cycle. All these amino acids are, with the exception of Gly, directly linked to non-amino acid molecules that are their precursors. (Although the biosynthetic pathways leading to Phe and Tyr and to His are directly linked to a non-amino acid precursor (Fig. 1), they seem too complex for an early evolution because they have at least ten biosynthetic steps in these pathways and so these three amino acids would evidently not fall within this classification (see Appendix)). As suggested by the extended coevolution theory, this might indicate that they were the first to originate during the evolution of the biosynthetic pathways of amino acids. (Gly is the only one of these amino acids that is not directly linked to one of these non-amino acid molecules of the glucose degradation pathway (Figs, 1, 2). Although the synthesis of Gly from Ser is well documented [9,44], the conversion of Gly to Ser also takes place normally [9,45]. For example, Gly is converted to Ser by reacting with formate in the presence of pyridoxal phosphate [9,45-47]. This favours the hypothesis that these two amino acids, Ser and Gly, were inter-convertible when these pathways originated).

If these were effectively the earliest amino acids to originate from non-amino acid precursors of the energetic metabolism pathways (Fig. 2) and if the main prediction of the extended coevolution theory is true, then all these amino acids (Fig. 2) should occupy a particular place within the genetic code table because they should be witnesses of the earliest phases of the evolution of the genetic code. Indeed, as other authors have observed [11], with the exception of Ser, all these amino acids (Fig. 2) are codified by codons of the GNN type. The distribution of these amino acids on these codons is not random and is obtained, by pure chance, with a probability equal to 3.9 × 10^-4 ^(see Appendix).

**Figure 3:**
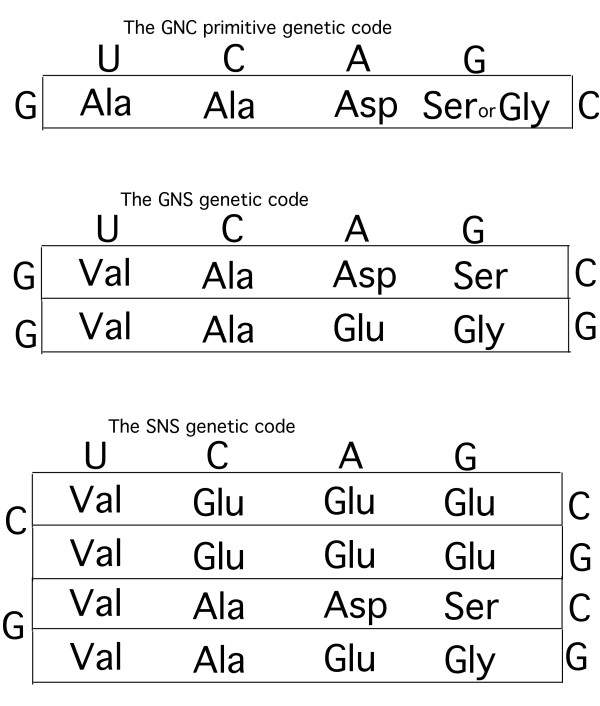
**This shows three stages of genetic code evolution.** All the abbreviations are standard. See text for discussion.

Therefore, this observation that the first amino acids to evolve along the biosynthetic pathways are the same ones that are mostly codified by codons of the GNN type leads us to suppose, in compliance with the extended coevolution theory, that there existed a type of primitive genetic code (mRNA) that possessed only the codons of the type GNC (or GNG) and codified only for the amino acids Ala, Asp and Ser or Gly (or Ala, Glu and Ser or Gly) (Fig. 3) from which the GNS code codifying for Val, Ala, Asp, Glu, Ser and/or Gly (Fig. 3) might have evolved. This is suggested by exploiting the results of Ikehara et al [48] who, for quite different reasons, suggested a genetic code origin that is, in some respects, similar.

It should also be borne in mind that as these amino acids are the most abundant in the experiments of prebiotic synthesis and in meteorites [40] they had already attracted the attention of researchers. Indeed, Eigen et al. [49] had suggested a primitive code with codons of the GNY type, which is partly compatible with what is maintained here, partly because it might be derived from a GNC code (Fig. 3) [50].

## Discussion

### Some comments on the evolution of the genetic code, as suggested by the extended coevolution theory

The evolution of the genetic code as suggested here needs some discussion and clarification.

**Figure 4:**
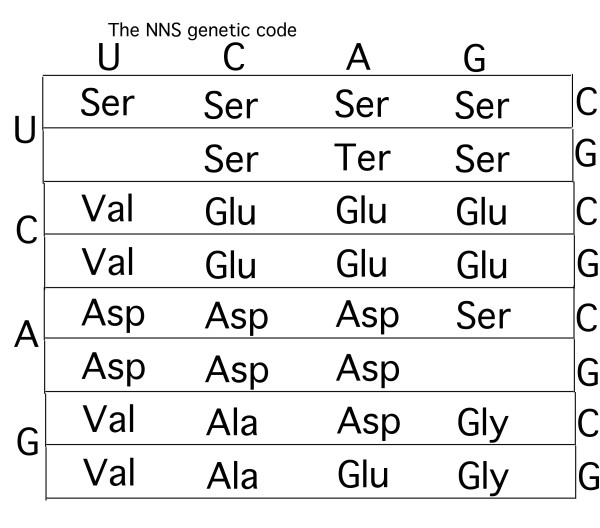
**This shows a stage of the evolution of the genetic code: the one in which the precursor amino acid codon domains are formed, as predicted by the coevolution theory**[9]**.** See text for discussion.

(i) Ser is not codified by any of the GNN codons whereas, on the basis of the considerations made here, it should be. However, the fact that Ser is biosynthetically inter-convertible with Gly [9,44-47] might indicate that Ser was codified by some or all the codons that today codify for Gly in the GNS and SNS codes (Fig. 3), and only with the NNS code (Fig. 4), i.e. when the codon domains of precursor amino acids were defined as predicted by the coevolution theory, did Ser cede some codons (GGS) to Gly (Fig. 4). This seems to be corroborated by the observation that, as Ser is also codified by AGY codons contiguous to the GGN codons of Gly, this might imply that the latter codons codified for Ser in a previous evolutionary stage.

From the evolutionary stage (shown in Fig. 4) of the genetic code on, the evolution of the code is fully described by the coevolution theory [9] (see Di Giulio and Medugno [35] for details on the entry times of amino acids into the genetic code).

(ii) The closer biosynthetic proximity between the pairs Ser-Ala, Ala-Val, Asp-Glu and Ser-Gly, as shown in Fig. 2, seems to find confirmation in the genetic code structure in that: (1) Ser-Ala and Ser-Gly have contiguous codons in the genetic code, i.e. they differ only in a single base, although Ser does not occupy the last row of the genetic code; (2) the pair Asp-Glu occupies the same box in the genetic code, i.e. their codons differ only in the third base and these amino acids are the same ones that, at the evolutionary stage of the biosynthetic pathways as indicated in Fig. 2, are more biosynthetically correlated; (3) the pair Ala-Val is part of the pyruvate biosynthetic family (Fig. 1) and their codons differ in only one base, a pyrimidine, even if these amino acids occupy the last row of the genetic code. All this seems to imply, in agreement with the extended coevolution theory, that amino acid pairs made in siblings by a non-amino acid molecule, i.e. the pairs Ser-Ala, Ala-Val, Asp-Glu and Ser-Gly (Fig. 2), the last of which might be in a precursor-product relationship [9], were particularly important in the earliest phases of genetic code origin because their organisation within the genetic code would also seem to reflect the closer biosynthetic proximity of these pairs (Fig. 2).

(iii) The here-maintained hypothesis that the amino acids that first evolved along the pathways of energetic metabolism (Fig. 2) formed the GNS code (Fig. 3) seems to rationalise why Asp and Glu are codified by GAN codons and not by ANN and CNN codons. Indeed, if the GAS codons had been attributed early on to Asp and Glu, they should have been both abundant on the first mRNAs and linked to them by a stronger historic constraint. Consequently, it would have been more difficult to concede them to product amino acids than the ANS and CNS codons making up the codon domain of Asp and Glu which instead must have been rare (see below) and also less historically constrained and, thus more easily transferable to the product amino acids, as seems to have happened. Therefore, this reasoning rationalises why Asp and Glu are codified by GAN codons and not ANN or CNN codons. Moreover, this strengthens the hypothesis of the existence of the GNS code for the very reason that Asp and Glu are codified by the GAN codons and not by some of those in ANN and CNN, as would have been more reasonable to expect considering the clearer biosynthetic relationship that Asp and Glu have with the product amino acids of their biosynthetic family compared to the less clear relationship they have with each other (Fig. 1). This should have resulted in a closer similarity between codons of Asp and Glu and codons of their product amino acids than with their own. The fact that this did not happen would seem to imply a very early involvement of GAN, or rather GNS, codons in genetic code origin because Asp and Glu are codified by these codons and not by those of the type ANN and CNN, as would instead be imposed by the clearer biosynthetic relationships with their product amino acids. In short, the codification of Asp and Glu by means of GAN codons might reflect the history of the very earliest phases of genetic code origin.

(iv) The evolution of mRNA as defined by the passage from the SNS (or GNS) code (Fig. 3) to the NNS code (Fig. 4) might have been highly facilitated if some codons were rarely used on mRNAs. In other words, let us admit that, for instance, there evolved in the SNS code: one or very few ANS codons codifying for Asp; one or very few CNS codons codifying for Glu: one or very few UNS codon codifying for Ser. It can be seen that in this way, all the precursor amino acid codon domains can be defined, i.e. the NNS code (Fig. 4), paradoxically without there actually being all their codons present. Indeed, it is sufficient for the first base of any one codon to be recognised, although read in triplets [51], in order to define the NNS code relatively fully. If the rarity of codons had been preserved in the evolutionary stages following the NNS codes (Fig. 4), then an amino acid precursor might have easily ceded part of its codon domain to the product amino acid without generating considerable translation noise in this transfer of codons. Naturally, every passage between the codes GNC (or GNG), GNS, SNS and NNS (Figs. 3, 4) must have been characterised by the rarity of the types of codons because the system was evolving and, for instance, the majority of tRNA molecules had yet to evolve, i.e. there existed very few types of tRNA molecule. In other words, it would seem that it is the very evolution of the code that implies codon rarity, allowing a faster and more efficient evolution by means of the mechanism of the coevolution theory. This leads us to suppose that the SNS form of code might have only partly preceded the NNS form because it would take just one codon, for instance of the ANS type, to define an entire codon domain and, therefore, an entire evolutionary stage of the genetic code. In other words, the evolutionary stage of the SNS and NNS codes might be less sharp than apparently shown in Figs. 3 and 4. Moreover, this indicates that the mRNA of the NNS code might have been much simpler than appears from the same Fig. 4.

(v) Exceptions to the "rule" of precursor amino acid codon domains seem to be the codons UUG (Leu) and AGG (Arg) (in white in Fig. 4), but also the codon AGC (Ser) although the latter might be derived from codons attributed to Gly, as suggested by Wong [9], but in any case outside the domain of Ser (Fig. 4). In other words, the codons UUR and AGR are the only exceptions observed in the precursor amino acid codon domains because they do not biosynthetically belong to the codon domain of the precursor in which they reside. However, while the codons UUR (Leu) might have been captured with a secondary mechanism by the codons in Ser's domain, for the AGR codons (Arg) there might exist a fascinating explanation. It is possible that the AGR codons of Arg derive from the codon domain of Asp and not from that of Glu, which is the natural precursor of Arg (Fig. 1) in that Asp intervenes in one of the terminal steps of the biosynthetic pathway of Arg [14,16]. Therefore, for Arg, the CGN codons might derive from the codon domain of Glu via ornithine or citrulline [16], while the AGR codons might derive from the codon domain of Asp [14,16]. This might therefore be an extremely interesting case of a double entry of an amino acid in the genetic code through two different amino acid precursors, something which has also been hypothesised for Ser [9]. This would provide a strong corroboration for the mechanism by which amino acids enter the genetic code, as suggested by the coevolution theory.

Finally, the CUS codons of Val (Leu) also apparently belong to the codon domain of Glu (Fig. 4). This might corroborate the hypothesis that these codons were ceded from Glu to Val. Indeed, the early phases of the evolution of NNS codes are characterised by codification limited to only six amino acids (Fig. 4) and therefore, the relative biosynthetic relationships might have made the amino acids Val and Glu biosynthetic siblings (Fig. 2). Although not entirely free of criticism, this viewpoint cannot be categorically excluded.

Nevertheless, there seems to be a much simpler interpretation provided by the SNS code (Fig. 3). Indeed, if in this evolutionary stage all the SUS codons codified for Val (Fig. 3) there would not have been any need for a real transfer of codons from Glu, but this might have only depended on the passage from the GNS to the SNS code provided that the SUS codons continued to codify for Val (Fig. 3).

## Conclusion

The coevolution theory [9] does not give a complete description of genetic code origin as it seems not to consider that the biosynthetic pathways of the amino acids that first entered the genetic code were important in the earliest phases of the origin of the code itself [9,40,52]. Whereas, with the extended coevolution theory it can be seen that there might have existed a GNC or a GNG code, but almost certainly a code of the GNS type, because the amino acids codified by these codons are in a clear biosynthetic relationship by means of their precursor non-amino acid molecules (Fig. 2) at the head of the amino acids' biosynthetic pathways and, therefore, must have characterised the earliest phases of genetic code origin.

The extended coevolution theory explains the existence, in the genetic code, of the pairs Phe-Tyr, Val-Leu and Thr-Met which are not in a clear biosynthetic relationship of precursor-product amino acids [14], by means of mere biosynthetic proximity. This is because, as the ancestral biosynthetic pathways take place on tRNA-like molecules, they enabled these biosynthetically close amino acids to have similar codons [16]. This cannot be achieved satisfactorily by the coevolution theory. For the sake of clarity and completeness, see also the comments already made on these amino acid pairs [16].

The coevolution theory [9] does not explain the presence of the codons of the amino acid pair Phe-Tyr inside Ser's codon domain (Fig. 4), whereas the extended coevolution theory explains its existence in this very domain through the mere biosynthetic proximity of the pathway leading to the synthesis of Phe and Tyr to that of Ser (Fig. 1).

Finally, the coevolution theory is unable to explain why Ala has codons contiguous to Val, even if it is clear that these two amino acids are biosynthetically correlated in that they are derived from pyruvate (Fig. 1). This theory even puts Ala and the Val-Leu pair in biosynthetically different domains [9,40], which seems to be mistaken. The extended coevolution theory, on the other hand, explains the relationships between these amino acids derived from the same non-amino acid precursor with the hypothesis that their ancestral biosyntheses took place on correlated tRNA-like molecules that allowed these amino acids to have likewise correlated codons in the genetic code [16].

## Appendix

It is necessary to calculate the probability with which the amino acids Ser, Gly, Ala, Val, Asp and Glu can be observed in the GNN codons of the genetic code while also taking into account the distribution of the amino acids in the non-GNN codons. Fisher's exact test seems to be able to calculate this probability. If we consider that, of these 6 amino acids, only Ser is not codified by GNN type codons, we obtain for amino acids with non-amino acid precursors: (i) 5 of these are codified by GNN codons (= a), while (ii) only 1 (Ser) is codified by non-GNN codons (= b). For amino acids with amino acid precursors, we have: (i) 0 of these are codified by GNN codons (= c), and (ii) 14 of these are codified by non-GNN codons (= d). By applying Fisher's exact test we obtain a probability P = 3.9 × 10^-4 ^(a = 5, b = 1, c = 0, d = 14) which is highly significant.

However, it could be objected that Val is 4 biosynthetic steps away from pyruvate, while Gly is not directly linked to PGA (Fig. 2) and therefore might not fall within the class of amino acids that evolved early on. To answer these strongly dubious questions, certain checks can be carried out.

Eliminating Val and Gly because they might not have entered the genetic code early on from the biosynthetic pathways' point of view (Fig. 2), we have P = 0.0035 (a = 3, b = 1, c = 0, d = 16). Therefore, under this hypothesis too, which actually seems extremely restrictive, we obtain a highly significant probability. Eliminating only Val (because Gly might have evolved very early on through interconversion with Ser [9,44-47]) or eliminating only Gly because Val is derived directly from pyruvate in a number of biosynthetic steps that, in qualitative terms, evolved rapidly and are not even numerous, we obtain a P = 0.0010 (a = 4, b = 1, c = 0, d = 15) that is still highly significant. In conclusion, these amino acids (Fig. 2) seem to have correlated GNN codons because they evolved early on in the ancestral biosynthetic pathways.

Finally, if we consider that His and Phe-Tyr are also derived from non-amino acid precursors (Fig. 1), we obtain P = 0.0081 (a = 5, b = 4, c = 0, d = 11); If we remove Val or Gly we obtain P = 0.014 (a = 4, b = 4, c = 0, d = 12); whereas, if both Val and Gly are removed, we obtain P = 0.031 (a = 3, b = 4, c = 0, d = 13). These probabilities indicate that considering His and Phe-Tyr as amino acids deriving from non-amino acid precursor does not substantially alter the results of the statistical test.

## Competing interests

The author declares that they have no competing interests.

## Authors' contributions

The author made all contributions. The author read and approved the final manuscript.

## Reviewers' Comments

### Reviewer's report 1

Rob Knight, University of Colorado, Boulder CO, USA

#### Reviewer Comments

This manuscript addresses an important question: whether there are traces in the pattern of codon assignments in the modern genetic code of its expansion from an earlier form, perhaps with simpler amino acids. The author addresses this problem from the perspective of his extensive previous work on the coevolution model, which argues that primordial genetic codes used simple amino acids that are produced in prebiotic syntheses and are encoded in the modern genetic code using codons beginning with G. For example, in previous work, he argued that the GNN-encoded amino acids Asp and Glu were early entries into the genetic code, and that the non-GNN-encoded amino acids Asn and Gln arrived later, in part because of the distribution of the metabolic pathways producing them and in part because of the fact that in some organisms they are produced by tRNA-dependent transamidation rather than by direct aminoacyl-tRNA synthesis. In the present work, he elaborates on this theory by adding constraints on the simplest amino acids, and presents statistical evidence that supports the idea that this type of coevolution shaped the modern genetic code.

[Author's Response]

No reply.

#### Reviewer Comments

The main issue I have with the present version of the manuscript is that it dismisses or fails to discuss other patterns in the genetic code for which the statistical evidence is at least as good as that presented here. This is not to say that the manuscript fails to cite prior work adequately: for example, the discussion of the development of the coevolutionary theory in this manuscript is very complete, and provides a nice self-contained introduction to interested readers. However, I believe that the contention that "we come to the conclusion that there can no longer be any doubts on the hypothesis that the origin of the organisation of the genetic code was affected by the biosynthetic pathways of amino acids." is overstated given that all the cited literature in support of this hypothesis is the work of the present author. Similarly, the statement on page 3 "The first is obtained by means of an extreme interpretation of the stereochemical hypothesis of genetic code origin which suggests that the genetic code originated because its organisation is somehow constrained by the stereochemical relationships between codons or anticodons and amino acids. This extreme interpretation seems totally absurd to me." does not adequately address the mounting statistical evidence from several laboratories, especially the Yarus lab, that there is a relationship between coding triplets and modern codon assignments that should not be ignored (although it is possible that future research will provide some reason why this observation is an artifact of some currently unsuspected process). Similarly, a long list of investigators including David Haig, Laurence Hurst, Stephen Freeland, David Ardell, Guy Sella, etc. have found evidence that the genetic code is error-minimizing compared to other possible genetic codes. I believe that, to be useful, new work on the genetic code needs either to embrace these patterns and explain them, or to argue against them on some grounds other than personal incredulity. After all, if the structure of the natural world were intuitively obvious we wouldn't need the scientific method, and it's important to take all the available data into account.

[Author's Response]

This paper presents a modification of the coevolution theory. I do not discuss other theories on the genetic code because, paradoxically, this is not 'the right place'. Even if other theories are well corroborated by evidence, I feel that my paper deals with such a specific issue – as the reviewer also acknowledges – that it 'rules out' comments on the other genetic code theories. On other occasions, I have not failed to tackle the problem raised by the referee [55,56] (see also below)).

The reviewer is making a serious claim: namely, that the biosynthetic relationships between amino acids are not in relation with the organisation of the genetic code. For instance, if we apply Fisher's exact test to the five biosynthetic families of amino acids [17], as the reviewer suggests, and then combine the five probability values in a single value, we obtain a highly significant probability (χ^2 ^= 34.8, df = 10, P < 10^-3^) (data not published). Ronenberg et al [14] also make a bitter criticism of the coevolution theory but, more generally, acknowledge that amino acids in a biosynthetic relationship have codons beginning with the same first base [14].

It is absolutely untrue that all the literature cited on this point is only my own. I have cited no less than 14 papers by other authors [5-13,16-22,34,37] which establish a relationship between the genetic code and the biosynthetic pathways of amino acids.

My suggestion refers to an extreme interpretation of the stereochemical theory, i.e. that if the origin of the genetic code were to start again from scratch, we would observe – according to this interpretation – the same assignments in the genetic code that we observe today. It is this extreme physicochemical determinism that seems so completely absurd to me.

A different argument regards the less extreme interpretations of the stereochemical theory. I have never neglected this theory (see, for instance reference [31]) and I have made it compatible with the coevolution theory [57], but I do not believe in the stereochemical theory because none of the presented evidence is, in my view, stronger, more important or more corroborative than the molecular fossils reported in Tab. 1 (see also replies to Reviewer 3).

All this evidence is compatible with the coevolution theory (see, for instance, reference [31] and replies to Reviewer 3).

My convictions are not based on grounds of personal incredulity but on molecular fossils (Tab. 1) which are 'eye witnesses' of the mechanism that gave origin to the genetic code (see also replies to Reviewer 3). If a different and credible interpretation of these molecular fossils were available, I would instantly renounce my convictions. There is nothing truly personal and unscientific in all this. I have taken all the data into account in Di Giulio [57].

#### Reviewer Comments

Similarly, it is not clear to me why the first amino acids to enter the code would be expected to be derived from other molecules that were not amino acids. If we assume that the genetic code arose in proto-cells that already had fairly sophisticated metabolism, e.g. the "RNA world" stage accepted by many researchers, it is less clear why pre-protein metabolism would not have generated a range of amino acids prior to genetic coding. Perhaps this point could be elaborated upon? For example, Eors Szathmary argues in the coding coenzyme hypothesis that we might expect complex amino acids to be introduced first, which is consistent with arginine's codon/binding site relationships demonstrated by myself and Michael Yarus.

[Author's Response]

I provide an extensive reply to this point with Reviewer 3. However, the Reviewer's question cannot find a simple answer because we do not understand the profound reason why the biosynthetic pathways of amino acids have to be in a relationship with the genetic code. Understanding this point might constitute the frontier research on genetic code origin [58].

It might have generated other amino acids, but their biosynthetic pathways seem to contain a quite different story than the one hypothesised by the Reviewer. Therefore, this point does not seem to require further elaboration (see also the replies to Reviewer 3).

It is possible: these are other heterotrophic interpretations on genetic code origin. In my view – and as I feel has been convincingly argued in this paper – the biosynthetic pathways of amino acids tell other stories (see also some of the replies to Reviewer 3).

#### Reviewer Comments

The calculation in the appendix used to calculate the significance of seeing 5 of 6 amino acids that have non-amino-acid precursors in the GNN codon block (binomial using n = 5, k = 5, p = 6/20) is definitely incorrect because it fails to take into account the distribution of amino acids in other coding blocks. I think the correct calculation, if we use 20 coding blocks for the 20 amino acids, is to say that 5 coding blocks are GNN-encoded, 15 are not, and these distribute into 5 GNN-encoded, no-precursor blocks, 0 GNN-encoded, precursor blocks, 1 non-GNN-encoded, no-precursor block (for Ser), and 14 non-GNN-encoded, precursor blocks. Using Fisher's Exact Test on these data, we actually get a P-value of approx. 0.00038: almost an order of magnitude more significant than reported. However, some caution about the space of possible coding blocks is warranted, and showing that the test holds over a range of these assumptions would be useful. The other statistics should also be re-done using this approach. However, it should be noted that the statistical significance of these results does not come close to that reported either for the stereochemical or adaptive theories of the code's evolution, so a more ecumenical view at this point would appear to be prudent.

[Author's Response]

I have changed the statistical test. Fisher's exact test provides much more significant results (see Appendix). I have also introduced some new observations (see final part of the Appendix).

I have conducted several tests in addition to those shown in the Appendix. I feel that Fisher's exact test is the one that really must be used to calculate these probabilities because it can take into account the structure of the genetic code by means of amino acids codified by GNN codons and by non-GNN codons. I thank the Reviewer for this suggestion.

The probability of 3.9 × 10^-4 ^(see Appendix) clearly indicates that the distribution of amino acids deriving from non-amino acid precursors is not in the least random in the genetic code. Even if this value is not close to the one associable to the stereochemical or physicochemical hypotheses, it nevertheless indicates that we have to explain it, which is what I have done in this paper.

I do not say that the stereochemical or physicochemical hypotheses are false, I simply explain what I observe using a prudent tone. Nevertheless, the stereochemical and physicochemical hypotheses cannot naturally explain what is observed in this paper.

#### Reviewer Comments

Finally, the conclusions seem to end rather abruptly with a discussion of specific product-precursor pairs. A more general concluding paragraph, including relationships between the present results and existing knowledge about the genetic code (including relationships to the adaptive and stereochemical patterns that have been shown using data from many laboratories) would be helpful for the general readership that Biology Direct attracts.

[Author's Response]

All the observations regarding the topic dealt with in the paper have been fully discussed. The paper is already over-long and its further extension with a discussion of the stereochemical and physicochemical hypotheses would be inappropriate in my view, partly because the observations reported therein are not easily reconciled with the stereochemical hypothesis, for instance, although there are models [57] that make the coevolution and the stereochemcial theories compatible. However, I feel that the Reviewer's suggestion is unsuitable as the paper does not aim to make a comparison with, for instance, the the stereochemical hypothesis. What the Reviewer suggests should be done elsewhere. Here I have introduced an extension of the coevolution theory and have not dealt with its relations with other theories.

### Reviewer's report 2

Eugene Koonin, National Institutes of Health, Bethesda MD, USA

#### Reviewer Comments

This article strives to develop Wong's co-evolution theory to additionally specify the order of amino acid recruitment to the genetic code. The main salient observation is that, with the sole exception of serine, amino acids that are synthesized from non-amino acid precursors are encoded by GNN codons. These are supposed to be the first amino acids in the code. This is an interesting idea but I think it is based on certain assumptions that are not spelled out in the paper. First, I think way too much confidence is granted the original co-evolution theory. It is a viable explanation for some aspects of the evolution of the code but, to me, frozen accident + partial optimization for translational robustness work at least as well. Second, and somewhat more subtly, an important hidden assumption is that, at the stage of the code evolution, central metabolism was already in place, so that amino acid biosynthesis pathways evolved from central pathways. This is far from being obvious. From my viewpoint, a more sensible approach would be to assume that the first amino acids were those that are most readily produced abiogenically. Granted, this list significantly overlaps with Di Giulio's but there is also considerable difference, and I believe it matters.

[Author's Response]

I have dedicated entire sections to specifying the various assumptions and, in particular, the section entitled "the extended coevolution theory", so the Reviewers' comment appears strange.

There is nothing new in the fact that the coevolution theory is not generally appreciated, but we must say why. The frozen accident and partial optimisation for translational robustness are compatible with the coevolution theory [31] (see replies to Reviewer 3). I repeat that I am convinced of the substantial correctness of the coevolution theory because: (i) the biosynthetic pathways are linked to the genetic code [5-13,16-22,34,37] and (ii) molecular fossils (Tab. 1) are 'eye witnesses' of the mechanism that structured the genetic code.

I reply extensively to this observation with Reviewer 3.

If the main suggestion of the coevolution theory is true, then there was a coevolution between the biosynthetic pathways of amino acids and genetic code organisation, implying that at least the metabolism of amino acids evolved when the genetic code evolved. Therefore, it is not utterly absurd to imagine that central metabolism was already in place because the biosynthetic pathways of amino acids start from there. Furthermore, the metabolic complexity of the RNA world has also been discussed (see, for instance, references [42,43]).

There is an overlap between the first amino acids to evolve along the pathways of central metabolism and those that are more abundant in the prebiotic syntheses or in meteorites. We will have to see which interpretation is right. This point is extensively discussed with Reviewer 3.

#### Reviewer Comments

The paper is written in a manner that makes it hard to figure out what is actually new.

I think it is worth to clearly formulate the difference from the traditional coevolution theory.

[Author's Response]

The Conclusions section answers this question.

The difference is reported in the Conclusions and on p. 9.

#### Reviewer Comments

I think it is worth citing the following paper:

Trifonov EN. The triplet code from first principles.

J Biomol Struct Dyn. 2004 Aug;22(1):1–11

That presents a good overview of different approaches used to infer the order of aminoa cid appearance in the code.

[Author's Response]

I have introduced this reference [54].

### Reviewer's report 3

Paul Higgs, McMaster University, Hamilton, Ontario

Nominated by Laura Landweber, Princeton University, Princeton NJ, USA

#### Reviewer Comments

The coevolution theory of the genetic code is a detailed and well-developed theory that describes the build-up from a simple structure encoding only a few amino acids to the current canonical code. Several important aspects of this theory make a great deal of sense to me, but other aspects seem less well justified. I will try to indicate the problems as I see them.

[Author's Response]

On the basis of what is written in this review against the coevolution theory, I do not understand what are the aspects of this theory that "make a great deal of sense" to this Reviewer.

#### Reviewer Comments

Firstly, I would like to relate this theory to the RNA World hypothesis, which supposes that there was a time early in the history of life at which both genetic and catalytic functions in organisms were carried out by RNA molecules. The genetic code and the translation process are the most important pieces of evidence that convince me that there was an RNA World. The whole point of translation is to take information from the mRNA and use it to make a protein. Furthermore, rRNAs and tRNAs are essential in the translation mechanism. Thus it seems clear that RNA came before the origin of the code. Although your papers and those of Wong do not mention the RNA World explicitly, it seems to me that the coevolution theory is perfectly consistent with the RNA World idea. Would you agree?

[Author's Response]

At a certain evolutionary stage. RNA must have become the 'master' of protocellular activity and from this point on I agree with the Reviewer's RNA world. Indeed I have published papers on this very topic [59]. In this paper, the coevolution theory is discussed in the terminal phases of the RNA world. There are also other papers of Wong's and my own on this issue [1-4].

#### Reviewer Comments

In my view, the late stage of the RNA World was already rather complex. I envisage cells enclosed by lipid membranes within which a well-developed, RNA-controlled metabolism was operating. These cells must already have solved the basic problem of accurate replication of relatively long RNAs (like rRNA), and they must have had a reliable energy input that could be coupled to the synthesis of large numbers of RNA polymers. The genetic code would have originated inside cells of this nature.

[Author's Response]

I totally agree: the genetic code originated very late on in the origin of life. In my papers I have stressed this point (see for instance reference [3]).

#### Reviewer Comments

One thing that is not clear in this paper is whether the metabolic reactions discussed are supposed to be catalyzed by RNAs or proteins. In particular, Figures 1 and 2 show that the synthesis of the earliest amino acids is related to the glycolytic pathway and the citric acid cycle. Since these are the earliest amino acids in the code, there could be no genetically encoded proteins prior to this. Are you therefore proposing that the glycolytic pathway and the citric acid cycle existed in the RNA World and that all the steps in these pathways were catalyzed by RNAs? This does not seem impossible to me, but it is a strong assumption, because it supposes that proteins have evolved to take over all the same catalytic steps formerly catalyzed by RNAs without changing any of the steps in the metabolism. Since the theory seems to depend on this assumption, it should be stated clearly.

[Author's Response]

We have discussed that the main catalyst in the early phases of genetic code origin was constituted by peptide-RNA molecules, whose evolution resulted in peptidyl-tRNA like molecules and, therefore, in the origin of the genetic code [1-4]. Therefore, during genetic code origin, metabolic reactions were catalysed by covalent complexes of peptides and RNAs (see Background) also involved in the catalysis of the glycolitic pathway and the citric acid cycle (see also below). If peptides were already involved in the peptide-RNA complexes, as I maintain, then the problem that the Reviewer raises is non-existent because the protein component already present should eventually prevail – without violating the principle of evolutionary continuity – over the RNA component. These ideas have been presented in several papers ([1-4,59], and need not be repeated here. An entire section "Why the genetic code originated" introduces these ideas so I do not feel any more need be said. I remind the Reviewer that very early catalysts could have catalysed reaction classes and, therefore, there might have been a very small number of enzymes [60].

#### Reviewer Comments

At the end of the results section, you touch on the fact that the earliest amino acids in the code are the most abundant in meteorites and in prebiotic synthesis experiments. We have recently considered this question in detail [61]. By combining measurements from several meteorites, experiments on atmospheric discharge, hydrothermal vents, icy dust grains and others, we show that there is a consensus of which amino acids are easiest to form non-biologically. Our analysis shows that ten amino acids are found widely in these cases, and that these can be ranked in decreasing order of frequency as Gly, Ala, Asp, Glu, Val, Ser, Ile, Leu, Pro, Thr. The other ten biological amino acids are not found in these non-biological situations. We consider our analysis to be strong support for certain aspects of the coevolution theory. We refer to the ten listed amino acids as 'early', because we suppose these were the first incorporated into the code, whereas the other ten are 'late' because they could only have been incorporated after the evolution of biochemical synthesis pathways. The early amino acids that emerge from our analysis are almost exactly the same as those taken to be early in the coevolution theory. Furthermore, Trifonov [54] has also carried out a ranking procedure that predicts a very similar order.

[Author's Response]

These observations are partly consistent with the coevolution theory. However, Ile, Leu, Pro and Thr are product amino acids according to the coevolution theory and therefore appeared late on, while they are early according to the observations of Higgs and Pudritz [61]. Nevertheless, it is unclear whether these amino acids entered the genetic code through the biosynthetic pathways or through their availability in the environment (heterotrophic origin) (see also below). Whereas, in order to explain the codification of these amino acids by GNN codons by means of the scheme in Fig. 2, the extended coevolution theory need only add that the glycolytic pathway and Krebs' cycle were already operative. I have introduced a reference for Trifonov's works [54].

#### Reviewer Comments

Although there seems to be general agreement about the distinction of early and late amino acids, our results make it clear that there are many different ways to make the early amino acids. These are easiest to form because they are thermodynamically least costly ([61]. In turn, this suggests that biochemical pathways might not be relevant at the earliest stages of genetic code evolution discussed in this paper. If these early amino acids were synthesized non-biologically, they might have been frequent in the environment and could have been used directly without requiring synthesis in the organism (heterotrophy). Alternatively, if they were synthesized by the organisms, the fact that they are easy to make suggests that many different reaction pathways would be possible, and that the pathways that were used in the RNA World may not be related to those used today, thus casting doubt on the relevance of Figure 2.

[Author's Response]

I do not understand why the ease of thermodynamic synthesis of these amino acids should not have been exploited by the biosynthetic pathways of amino acids. It should rather be expected that the thermodynamic opportunities be exploited biologically. It is unclear why these amino acids should not be able to coincide. It is also unclear why the pathways used by the RNA world should be different from those used today. These pathways are fundamental and, once they were acquired it would have been difficult to change them. Does the Reviewer think that the metabolism of the RNA world was different from that of today? Why? And, in particular, why should the biosynthetic pathways of amino acids be different from those of the RNA world? Although minor changes are to be expected, the majority of the pathways present in the RNA world should have been preserved unchanged even in the later evolutionary stages. It must be borne in mind that most of the pathways in Fig. 1 evolved in a world in which the catalytic component might have already been represented, albeit partly, by proteins (peptides), at least those that were codified at that time, made up of only the amino acids in Fig. 2. Complexes of peptide-RNA catalysts, some of which were of heterotrophic origin, might also have been used for the syntheses in Fig. 2 with the additional condition that the first amino acids were codified only through the biosynthetic pathways in Fig. 2. However, the key point is that the amino acids in Fig. 2 are the same ones codified by GNN codons, and this association is statistically highly significant. If, more generally, we consider that the biosynthetic pathways of amino acids are linked to genetic code organisation [5-13,16-22,34,37], then the relation between GNN codons and Fig. 2 becomes highly significant and might truly explain the very earliest phases of genetic code origin.

#### Reviewer Comments

One interesting point of agreement is that the five most frequent amino acids in our list (Gly, Ala, Asp, Glu, Val) are exactly those coded by GNN codons, and this suggests something very similar to that shown in your Figure 3. In your results section, you mention GNN codons, but then say that there was a primitive code that possessed only codons of the type GNC or GNG from which the GNS code developed (where S = G or C). I do not understand the reason that the third position base was restricted to G or C. I would suppose that the wobble pairing at the third position in the anticodon-codon interaction is a fundamental aspect of RNA structure that would have been the same in the earliest tRNAs, *i.e*. I would assume that a tRNA with wobble base G could pair with codons ending C or U, and that a tRNA with wobble base U could pair at least with codons ending A and G (as occurs with most bacterial tRNAs today), and possibly with all four bases at the third position (as occurs with most mitochondrial tRNAs today). In other words, I think that the two-codon and four-codon boxes seen in the modern genetic code arise naturally from the properties of RNA structure and that these would also have occurred in the earliest code. In contrast, wobble pairing does not occur at first position, so there is no problem with having the first position restricted to G or C, as in Figure 3.

[Author's Response]

The GNC code was suggested by Ikehara et al. [48] and I make use of their results. The truly important point is that the biosynthetically early amino acids are codified by GNN codons. It is irrelevant which form of code, for instance GNS, GNR or other, was actually operative. However, the reason might be that, as all the codons start with G, it might have resulted in a general enhancement of G and C in mRNAs and it is also for this reason that the scheme in Fig. 3 was chosen. I also prefer the GNS codon because I believe that this took place at a very high temperature, thus favouring RNAs rich in G and C [3]. Therefore, I partly agree with the Reviewer. More generally, I must say that this is not an important point and should not be overstressed.

However, Eigen et al. [49] also prefers codons starting with G (GNY). I prefer restricting the third codon position to just two bases because it is thus easier to achieve the evolution of mRNA [3,4].

#### Reviewer Comments

A key point of the coevolution theory is that, when a new amino acid is added, it takes over some of the codons previously assigned to its precursor. If all the amino acids in the current code are traced back to their earliest precursors, then we arrive at Figure 4 (or something similar, if we interpret 'C' and 'G' at third position as 'U or C' and 'A or G'). This code arises naturally from the logic of the coevolution theory, but there is no direct evidence for it, *i.e*. this is a prediction of the theory and not a basis for it. It does not follow on as an obvious step from the GNN code in a predictable way. There seems to be no reason why this rather bizarre pattern of placement of the earliest amino acids should have occurred. I find Figure 4 strange because it sets some difficult challenges for molecular recognition during the assembly of amino acyl-tRNAs. In particular, the shapes of the codon domains occupied by Asp, Glu and Ser are complicated. Presumably there were RNA catalysts that carried out the job of current amino acyl-tRNA synthetases. For correct charging of tRNAs, these synthetase RNAs would have had to distinguish large sets of tRNAs from one another, possibly by recognition of the bases in the anticodon. The anticodons for Asp tRNAs, according to Figure 4, would be GAU, UAU, GGU, UGU, GUU, UUU, and GUC (it should be remembered that pairing is antisense, so the first anticodon base pairs with the third codon base). To recognize this combination of anticodons would require some complex mixture of logical operations combining bases at all three anticodon positions, for example: IF (3rd base = U AND 2nd base C) OR (3rd base = C AND 2nd base = U AND 1st base = G) THEN charge with Asp. This would either require a very complex recognition process for a single synthetase, or it would require separate synthetases for each codon block that would carry out the same reaction of charging the tRNA with Asp. Neither of these options seems simple or parsimonious. The same would be true for other amino acids in this arrangement of the code.

[Author's Response]

The Reviewer is mistaken. There is direct evidence from the genetic code indicating, for instance, that the majority of ANN codons codified for Asp. Therefore, it is the biosynthetic relationships reflected in the genetic code that define Fig. 4. This is a prediction of the coevolution theory but it is also supported by the distribution of the biosynthetic pathways of amino acids in the rows of the genetic code (see, for instance, Taylor and Coates [11]).

Whereas, there is an obvious step which derives Fig. 4 from the GNN code. This consists of the fact that, once the codifications were assigned to the first six amino acids on GNN codons, it was necessary to immediately extend the meaning, as Crick also suggests [51], to many codons in the code, thus generating the code in Fig. 4. The Reviewer should read sections (iii) and (iv) of the Discussion more carefully. Fig. 4 is not strange and, in particular, the shape of the codon domain of Asp and Glu and Ser is linked to the rows of the genetic code which, as suggested by Taylor and Coates [11], are in relation with the biosynthetic families of these amino acids.

The coevolution theory does not necessarily envisage that the aminoacyl-tRNA synthetases were present at this stage of genetic code origin because the tRNAs might have been charged by means of the biosynthetic pathways of amino acids. There was no need for the aminoacyl-tRNA synthetases. The Reviewer's criticism is therefore weakened. Moreover, as stated in the paper, the mRNAs might have been simpler than Fig. 4 leads us to believe. See the subsections (iii) and (iv) of the Discussion, in which the complexity of recognition maintained by the Reviewer is considerably reduced.

#### Reviewer Comments

An alternative that I favour at the moment is that the GNN code developed into a 'four-column' code in which all codons in the same column coded for the same amino acid: NUN = Val, NCN = Ala, NAN = Asp (and/or Glu) and NGN = Gly. This is an obvious simple step from the GNN code: all we do is relax the restriction that the first position must be G. It is very simple for molecular recognition by the synthetases because only the middle anticodon base needs to be recognized. For example, a single synthetase that adds Val to all tRNAs with 2nd anticodon base = A would be sufficient to assign all NUN codons to Val. Furthermore, the four column code explains why amino acids with similar physicochemical properties end up in the same columns of the code whereas amino acids in the same row (same first codon base) do not have similar properties. The difference between rows and columns shows up clearly when we look at the rates of evolution of 1st and 2nd position sites in proteins and the variability of these sites among species [62]. According to this argument, it is physicochemical properties that are important in determining where new amino acids are added to the code. Amino acids will be added into positions that were formerly occupied by amino acids with similar properties because this is minimally disruptive to the proteins encoded by the code at the previous step. As an example of the difference between this argument and the coevolution theory, consider Ile, which is assigned to codons in the AUN box. According to the coevolution theory, Ile ends up in this position because AUN was originally Asp and Ile is synthesized from Asp. According to the physical property argument, Ile ends up in this position because AUN was originally Val and Ile is similar to Val. It is well known that neighbouring codons in the canonical code tend to specify similar amino acids and hence that the code seems to be optimized with respect to randomly reshuffled codes [63]. The physical property argument summarized above explains how the optimality of the canonical code arises as a result of its evolution from the four-column code. The coevolution theory ignores this issue.

[Author's Response]

It is not obvious, and it indeed does not seem sensible, why a column code specifying amino acids Ala = NUN, Asp = NAN (and/or Glu) and Gly = NGN should have been created. Why should such a code have been created? Whereas the opposite is obvious for the coevolution theory. It would be sufficient to insert, from the GNN code, the other amino acids on the columns, according to their physicochemical properties, without passing through this fairly useless code, partly because there is no evolutionary link between these five amino acids (Val, Ala, Asp, Glu and Gly) and the other amino acids that will occupy the columns; and if this link had been based on the physicochemical properties of amino acids, it would have been inefficient because, for instance, in the column NGN = Gly, there are the smallest (Gly) and the largest (Trp and Arg) amino acids. Indeed, although the physicochemical properties are linked to genetic code organisation, they are not highly minimised and so these properties might have played only a subsidiary role in genetic code evolution [31,56].

If, on the other hand, the column code was obvious, then the code in Fig. 4 would be equally obvious because it is organised in rows, as Taylor and Coates suggest [11] the genetic code is organised. The GNN code, which the Reviewer also accepts, is a row code and, therefore, the next step in genetic code evolution must 'necessarily' be a code organised in rows because it evolves on row constraints existing in its precursor (GNN code) and not the one organised in columns suggested by the Reviewer which implies a radical change in the logic for the construction of mRNA.

The problem of the synthetases is non-existent because, as already suggested, the coevolution theory can envisage the charging of tRNAs by means of the biosynthetic pathways of amino acids or, at least, can envisage a limited intervention of the synthetases.

The coevolution theory is compatible with the observation that the physicochemical properties of amino acids are better allocated on the columns of the genetic code. I have dealt with this issue extensively, see for instance Di Giulio [31].

Whereas, from the viewpoint of the coevolution theory, it is the rows (biosynthetic pathways) that are important for determining where an amino acid will be added, with the columns deciding only the reduction in the translation noise compatibly with the row allocation.

The coevolution theory considers this aspect. If we consider that, according to the extended coevolution theory, the GNN code preceded the current code then, as the amino acids evolved along the biosynthetic pathways organised in rows, the amino acids were allocated in columns in an attempt to reduce the physicochemical distances between amino acids [31]. If hydrophobic amino acids were allocated on a given column of the code (first column), then the majority of hydrophobic amino acids biosynthetically originating in the various rows would have the possibility to be allocated on the code's first column, and so on. In other words, the coevolution theory is perfectly compatible with the distribution of the amino acids' physicochemical properties in the genetic code [31].

#### Reviewer Comments

The amount of credence that one gives to the coevolution theory depends on the extent that one believes that amino acid synthesis occured on tRNAs. There are two cases where the evidence for this is very strong: Asp Asn and Glu Gln. These reactions occur on the tRNAs in both Archaea and Bacteria, as shown in Table 1, and Di Giulio [33] is cited as evidence that these are molecular fossils. The Ser Cys case would be another good example, but it is only found in Archaea, according to the table. The cases of Met fMet and Ser Sec are less relevant because they involve non-standard amino acids that do not have their own codons. It would be interesting to have more details on all the examples in Table 1. For any one of the examples, are all the enzymes that carry out this reaction homologous? This is particularly relevant if the function is shared by Archaea and Bacteria – it is necessary to argue that the sequence is homologous in the two domains in order to exclude the possibility that the function evolved independently. When one of these functions is present in a domain, is it present in the majority of species in this domain? This is important in order to rule out horizontal transfer of a gene from one domain to a small group of species in the other domain. As far as I know, Sec occurs patchily in unrelated groups of organisms, so even though the Ser Sec reaction occurs in all three domains, there is probably not a good case that it is ancestral. On the other hand, my understanding of the Asp Asn and Glu Gln cases is that these are really ancestral to the split of Archaea and Bacteria. Please could you summarize in more detail how strong the evidence is that all the cases in Table 1 evolved ancestrally to the split of the domains of life?

**Table 1:** The biosynthetic pathways that transform one amino acid into another when the transformation takes place on tRNAs and their phylogenetic distribution. See Sheppard et al [53] for further information.

Pathways	Phylogenetic distribution
Glu-tRNA^Gln^->Gln-tRNA^Gln^	Bacteria, Archaea and chloroplasts
Asp-tRNA^Asn^->Asn-tRNA^Asn^	Bacteria and Archaea
Ser-tRNA^Sec^->Sec-tRNA^Sec^	Bacteria, Archaea and Eucarya
Met-tRNA^fMet^->fMet-tRNA^fMet^	Bacteria and organelles
Ser-tRNA^Cys^->Cys-tRNA^Cys^	Archaea

[Author's Response]

I have dedicated an entire paper [33] in an attempt to establish whether or not the pathways in Tab. 1 are ancestral traits. The conclusion of this analysis [33] is that there is no reason why these pathways (Tab. 1) should be derived traits. I cannot summarise the contents of that paper in this work. I cite that paper [33]. Three other references [19-21], and not only my own, are cited and indicate that these pathways might be molecular fossils (see below).

However, if the Reviewer accepts the ancestrality of the pathways Asp->Asn and Glu->Gln, why then should he not accept the ancestrality of, for instance, Ser->Cys? Does he perhaps think that these pathways were generated by different mechanisms? This seems absurd to me. There is absolutely no chance of these five pathways (Tab. 1) evolving independently without any clear selective pressure (see below). It is better to think that these pathways are the expression of the same mechanism that produced them because they are 'homologues', i.e. they do the same thing.

The pathway Ser->Sec is certainly homologous, at least between Archaea and Eukarya [64,65], and so it should be extremely ancient. In Archaea and Eukarya, this pathway takes place in two steps, while it is in one step only in Bacteria [65]. However, all these enzymes are homologues, i.e. they share a common origin and so this pathways seems extremely ancient and is also very widespread, contrary to what the Reviewer says [66]. The pathway Ser->Cys has also been suggested as being present in the LUCA [67], However, the pathway Ser->Cys has only been found in a few archebacteria and so its phylogenetic distribution would seem to indicate, given its phylogenetic rarity, that it is a derived trait, as the Reviewer claims. Nevertheless, all these pathways (Tab. 1) are extremely difficult to evolve because they must necessarily create some intermediates, such as Ser-tRNA^Cys ^which, if they ended up on ribosomes would have disastrous effects on cell life. Therefore, these pathways are difficult to evolve. This and other arguments are reported in Di Giulio [33], which concludes that all these pathways are molecular fossils of the mechanism that established the genetic code. The Reviewer is referred to my reference [33]. Finally, why should these pathways have evolved recently to do the same thing that an aminoacyl tRNA synthetase did so well? Why? Why replace an aminoacyl tRNA synthetase with another synthetase whose first step would charge an amino acid on a tRNA specific for another amino acid (for instance Ser-tRNA^Cys^)? Why? I invite the Reviewer to write a paper on this issue, addressing these questions and those raised in Di Giulio [33]. The truth is that there are no answers to these questions and the only way that these pathways (Tab. 1) can be rationalised is to view them as ancestral traits. In conclusion, the evidence in favour of the ancestrality of these pathways is considerable (see [33]).

#### Reviewer Comments

If we accept for a moment that the Asp Asn and Glu Gln reactions evolved ancestrally to the split between the domains, the next question is whether these reactions were initially catalyzed by RNA or proteins. Asn and Gln are late additions to the code. A relatively diverse set of amino acids, such as the ten early amino acids listed above, could have been present in the code before Asn and Gln were added. Thus the first catalysts that carried out these reactions may have been proteins composed of the early amino acids. The fact that these reactions occur on tRNAs does not necessarily mean that they were relics of the RNA World. Similarly, the fact that these two amino acids are synthesized on tRNAs does not necessarily mean that the metabolism of the earliest amino acids in Figure 2 occurred on tRNAs.

[Author's Response]

I do not understand the relevance of catalysts in these reactions. They could have been RNAs, peptide-RNAs or even proteins composed of early amino acids. I favour catalysis by peptidyl-tRNAlike molecules as the true catalysts at this stage of the genetic code [3,4]. This excludes nothing. These pathways have all the requisites to be molecular fossils of the RNA world ([31,33,68]).

If, as the Reviewer suggests, we accept that Asp->Asn and Glu->Gln are ancestral, then along with the other pathways (Tab. 1), this would strongly corroborate the coevolution theory. But this theory says that precursor-product transformations took place on tRNAs. However, why should only these transformations of amino acids take place on tRNAs? Evidently the implication is that the entire metabolism, or at least that involving all amino acids, took place on tRNAs and, therefore, the pathways in Fig. 2 could also take place on tRNAs. This conclusion is also suggested by other authors [28,31,41-43]. This is the extended coevolution theory. With this assumption, all the weaknesses of the coevolution theory are removed.

The fact that these two amino acids are synthesised on tRNAs does not necessarily mean that the metabolism of the amino acids in Fig. 2 took place on tRNAs, but all the pathways in Tab. 1, one of which takes place in two steps, might imply, more generally, that metabolism took place on tRNAs. Furthermore, Glu from Glu-tRNA^Glu ^intervenes in the biosynthesis of chlorophyll [31] and this, together with other observations on a more general role of aminoacyl-tRNAs in metabolism [69] might strengthen the hypothesis that the entire metabolism took place at this stage on tRNAs [31,41-43].

#### Reviewer Comments

If I understand correctly, all five examples in Table 1 are single step reactions catalyzed by a single enzyme. This is also a good reason to suppose that synthesis on the tRNAs is a relevant mechanism of synthesis for these amino acids in particular. However, most of the late amino acids with the exception of Asn and Gln have very long synthesis pathways involving many intermediates (as shown in Figure 1). There seems to be no evidence that any of these were synthesized on tRNAs. There are eight steps shown from Glu to Arg, for example. It seems to be stretching the theory too far to suppose that there were eight sequential reassignments of Glu codons to intermediate molecules, that all these intermediates have completely disappeared again from the modern code, and that all these reaction steps that formerly occurred on the tRNA have now been replaced by equivalent steps that occur without the molecules being attached to tRNAs. It is simpler to suppose that the pathways to synthesize these late amino acids were never associated with tRNAs, and that the intermediates do not appear in the modern code because they were never added to the code in the first place. Thus, the evolution of the synthesis pathways is important in allowing the diversity of the code to build up, but this does not influence which codons are assigned to which amino acids. If synthesis does not occur on the tRNA, there is no reason why the product amino acid should take over the codons of its precursor. Since most of the late amino acids have long synthesis pathways, it is likely that they arose in the protein world, and that the steps were catalyzed by proteins made from earlier amino acids. The situation may be different for Ile, Leu, Pro and Thr, which occur non-biologically and are thus included among the early group in our ranking, although they are less frequent than the simplest amino acids. These four also have relatively long synthesis pathways on Figure 1. These may have existed in the environment at the time the code originated if their rates of non-biological synthesis were high enough, or they may have been synthesized by RNA-catalyzed pathways. In either case, the protein enzymes catalyzing the pathways on Figure 1 would have evolved later and there is no reason to suppose that these pathways are the same as those that existed when these amino acids were added to the code.

[Author's Response]

No. In the Ser->Sec pathway there are two biosynthetic steps. Absolutely not. As stated above, the first step regards the charging of an amino acid on a tRNA specific for another amino acid, which is a very dangerous hybrid because, if it ended up on the ribosomes it would be lethal. Therefore, there is no 'good reason' why these pathways should be used to synthesise these amino acids [33].

The fact that Ser->Sec takes place in two steps seems to indicate that this was possible, contrary to what the Reviewer maintains.

The intermediates that are not amino acids would not appear in the code. Only amino acids should appear in the evolving code according to the coevolution theory.

There is nothing strange in this. If biosyntheses took place on tRNAs then, when the code was completely developed, a strong selective pressure would have been triggered to remove tRNAs from metabolism because the tRNAs were extremely cumbersome and it is therefore not surprising that today we only observe the relics of these events (Tab. 1) [31].

Today it might seem true that syntheses on tRNAs were inefficient. Nevertheless, this is the very story that these fossils tell: synthesis on tRNAs.

However, there is evidence – again from molecular fossils – that biosynthetic pathways might have taken place on tRNAs. the pathway to His starts with a reaction producing N'-5'-phosphoribosyl-ATP, which is held to be a fossil of RNA [43,70].

The fact is that the biosynthetic pathways are linked to genetic code organisation [5-13,16-22,34,37] and therefore give credence to syntheses on tRNAs.

In several biosyntheses of amino acids, there are amino acids as intermediates. This implies, in agreement with the coevolution theory, that these could have been incorporated into the evolving code but were subsequently substituted [9,16]. However, I see no problem here with the coevolution theory, even if the biosyntheses were catalysed by proteins.

That the Reviewer's suggestion regarding the amino acids Ile, Leu, Pro and Thr is probably false and that, more generally, the Reviewer's entire argument regarding both the very early amino acids and the 'column code' is dubious, is demonstrated by the fact that the 'system' that led to the GNN code must have been extremely efficient because it was able to achieve a clear classification of amino acids only on the basis of their frequencies, separating them into two groups: Gly, Ala, Asp, Glu and Val in the GNN code, and Ile, Leu, Pro and Thr. Evidently an extremely efficient system! Is it possible that the system was able, only on the basis of frequencies, to incorporate into the GNN code only the first amino acids in the ranking of Higgs and Pudritz [61] without making an error and that the same system created the column code by extending the codification of these amino acids? This seems absurd because in the first phases there seem to be strong stereochemical constraints while, in the column code, these constraints are completely relaxed. Is this possible?

Why should the pathways be different? I have already answered this observation. It is better to maintain an old pathway if only to maintain evolutionary continuity. I fail to understand why the change of catalysts should entail a change of pathway even if, as already suggested, the majority of steps in Fig. 1 were catalysed by peptide-RNA complexes. (Finally, the coevolution theory does not clearly define the early phases of genetic code origin, i.e. the GNN code, because it considers that the precursor amino acids Gly, Ala, Val, Ser, Asp and Glu entered the code without following the biosynthetic pathways. The Reviewer adopts a similar standpoint in which the amino acids Gly, Ala, Val, Asp and Glu entered the GNN code without using the biosynthetic pathways. The extended coevolution theory has a different interpretation: the amino acids Ser, Gly, Ala, Val, Asp and Glu entered the code through the biosynthetic pathways. Therefore, the question is as follows: why should the amino acids Ile, Leu, Pro and Thr, which appear in prebiotic syntheses and are early in the ranking of Higgs and Pedrutz [61], not have been added to the code as the amino acids codified by the GNN codons, but entered the code through the biosynthetic pathways, as the coevolution theory suggests? This would constitute a difficulty for this theory because all these amino acids were present in the prebiotic environment and it would not be clear why some entered the code directly while others entered via the biosynthetic pathways. The extended coevolution theory removes this difficulty as it treats all amino acids in the same way: they all entered the code via the biosynthetic pathways. As already suggested, the Reviewer's hypothesis presents some inconsistencies. Why should only the amino acids Gly, Ala, Asp, Glu and Val have been codified by GNN codons while the other amino acids (Ile, Leu, Pro and Thr) were added to the code later on? Did this choice take place only on the basis of frequency? It seems to me that the frequencies are too weak a constraint to explain the clear distinction between these two groups of amino acids while the biosynthetic pathways seem to be a sufficiently strong constraint to explain these observations consistently with the allocations of all these amino acids in the code.)

#### Reviewer Comments

I have thus argued that while the case for the reactions Asp Asn and Glu Gln occurring on tRNAs is very strong, this cannot be generalized to other amino acids. Without this generalization, the central importance of precursor-product relationships in the coevolution theory breaks down. These two cases fit with the argument based on physicochemical properties and four-column code as well. If NAN codons were initially Asp and Glu, then it makes sense that Asn and Gln would also be added in this column because they are more similar to Asp and Glu than they are to the amino acids in the other columns. Thus the theories agree for these two cases.

[Author's Response]

All these pathways (Tab. 1) must be expressions of the same mechanism that generated them because believing that they might be derived from different selective pressures is absolutely absurd as no selective pressure can be clearly identified as having generated them [33]. Hence, if we accept the case of Asp->Asn and Glu->Gln then this must have been generalised among the other amino acids and, thus, the coevolution theory is strongly corroborated.

It is incredible how the Reviewer can say that the pathways on tRNAs involving the transformations Asp->Asn and Glu->Gln, which are a direct prediction of the coevolution theory, are also 'in agreement' with the columns theory for the simple reason that Asn and Gln are more physicochemically similar to Asp and Glu, respectively. Let's be serious: the two pieces of evidence are clearly different in quality and, therefore, the two theories are not at all equal on this point. This is because, if pathways on tRNAs, Asp->Asn and Glu->Gln would be historic evidence and hence of extraordinary importance in understanding the origin of the genetic code [33]. Whereas, the physicochemical similarity between these pairs of amino acids should have played only a secondary role in allocating Asn and Gln to the columns, as also predicted by the coevolution theory because it would be the necessary consequence of these pathways on tRNAs (see, for instance reference [31]). In short, although the two theories agree on this point, they receive different levels of corroboration: the coevolution theory is strongly corroborated by it, while the columns theory is not and it acquires only a subsidiary role.

#### Reviewer Comments

Finally, although any discussion of metabolic pathways in the RNA World is bound to be speculative, we can be much more concrete in discussing pathways in modern organisms. Figures 1 and 2 are presented as 'the' pathways for amino acid synthesis, but I presume these are based on a particular organism like *E. coli*. I do not know to what extent these pathways are truly conserved between all species. Has anyone carried out this analysis using sequence data from complete bacterial and archaeal genomes? I would be interested to know what fraction of these complete genomes contains an enzyme for each of the steps in Figure 1. If enzymes do not exist for these steps, are there alternative synthesis pathways, or are the organisms reliant on taking in these amino acids as food? In general, are pathways of amino acid sequences in modern protein-based organisms more conserved than some other pathways that might be considered to be less essential to cell function? If the pathways are not conserved in modern organisms, the chances that they would be conserved as far back as the RNA World are slim.

[Author's Response]

If logical-evolutionary analyses were conducted on these pathways and it was concluded that these were molecular fossils [33], it would not necessarily be true that the metabolic pathways of the RNA world are only speculations ([43,68]).

The majority of organisms use pathways essentially similar to those of *E. coli *[43,71]. I have been following this literature for many years and it does not seem to me that there are significant deviations from the pathways presented in Figs. 1 and 2.

There have not been analyses of this type or they have been very limited and nevertheless confirm the scheme of the biosyntheses of *E. coli *[71].

However, the intimate relationship between the biosynthetic pathways of amino acids and the organisation of the genetic code is such as to make the research suggested by the Reviewer superfluous because it is not possible that this intimate relationship holds only for the biosynthetic pathways of *E. coli *and its genetic code.
